# Physicians’ Knowledge, Attitude, and Experience of Pharmacogenomic Testing in China

**DOI:** 10.3390/jpm12122021

**Published:** 2022-12-07

**Authors:** Tong Jia, Caiying Wu, Xiaowen Hu, Sicong Li, Xinyi Zhang, Yuchun Cai, Jing Chen, Luwen Shi, Christine Y. Lu, Xiaoyan Nie

**Affiliations:** 1 Department of Pharmacy Administration and Clinical Pharmacy, School of Pharmaceutical Sciences, Peking University, Beijing 100191, China; 2 International Research Center for Medicinal Administration, Peking University, Beijing 100191, China; 3 Department of Population Medicine, Harvard Medical School and Harvard Pilgrim Health Care Institute, Boston, MA 02215, USA

**Keywords:** pharmacogenomic testing, physicians, perspectives, implementation, China

## Abstract

(1) Background: As prescribers, physicians play a decisive role in applying and promoting pharmacogenomic (PGx) testing in clinical practices. So far, little is known about physicians’ perspectives on PGx testing in China. The aim of this study was to assess physicians’ knowledge of, attitude towards, and experience of PGx testing in China. (2) Methods: A 39-question online survey was developed. Participants were physicians recruited through two platforms, MEDLINKER and “Dazhuanjia”. (3) Results: A total of 450 respondents completed the survey and 366 questionnaires were eligible for analysis based on the inclusion criteria. Among all included physicians, 275 (75.1%) had heard of PGx testing before. More than half rated their knowledge of PGx testing as “Fair” (61.5%) while 20.0% chose “Excellent” or “Good” and 18.6% chose “Poor” or “Terrible”. “Guidelines, consensus, and treatment paths for disease diagnosis and treatment” (72.7%) were the most preferred sources of information about PGx testing. Respondents were confident in their personal capacity to conduct PGx, with an average score of 3.30 ± 0.09 (out of 5.00). Most respondents (75.6%) believed that PGx could “help to improve efficacy and reduce the incidence of adverse reactions”. Targeted cancer therapy (score 78.95 ± 1.26 out of 100) was considered the field where PGx testing had its highest value. Lack of professionals and knowledge (n = 186, 67.6%), high costs of testing (n = 170, 61.8%), and lack of hospitals to offer PGx testing (n = 166, 60.4%) were identified as the primary obstacles to increasing the uptake of PGx testing in China. Academic conference (n = 213, 72.4%) was considered the most efficient way for physicians to obtain information about PGx testing. (4) Conclusions: Physicians in China have poor knowledge about PGx testing; nonetheless, they generally had confidence in their capacity to order PGx testing and positive attitudes towards the use of PGx testing in routine clinical practices. Future efforts to promote the uptake of PGx testing should focus on foundational education and practical training.

## 1. Introduction

Interindividual variations in drug responses are commonly observed in all therapeutic areas. Despite that some nongenetic factors (such as gender, age, weight, pathophysiological condition, comorbidity, and drug interactions) can influence drug efficacy and toxicity, genetic variation is one main factor contributing to the individual differences in drug responses [1,2]. Pharmacogenomics (PGx) can elucidate inherited individual differences in drug response, thereby provide a scientific basis for optimizing personalized drug regimens in clinical practices. Pharmacogenomics has rapidly developed into a field with great popularity and application prospects. The benefits of some PGx testing in clinical practice have been demonstrated by recent randomized controlled trials [3,4,5]. Over 500 labels have been attached to PGx testing recommendations and black box warnings by the U.S. Food and Drug Administration (FDA) [6]. Guidelines developed by the Clinical Pharmacogenomics Implementation Consortium (CPIC), the Dutch Pharmacogenetics Working Group (DPWG), the Canadian Pharmacogenomics Network for Drug Safety (CPNDS), and the French National Network (Réseau) of Pharmacogenetics (RNPGx) also recommend applying a range of PGx testing in clinical practices [7].

Applying PGx testing in clinical settings can help to optimize treatment regimens, guide the rational use of drugs, and thus improve therapeutic outcomes by maximizing drug efficacy and minimalizing adverse drug reactions. Take the application of PGx testing on antiplatelet therapy as an example. PGx can help to identify patients with a moderate and low metabolism of CYP2C19 and reduce the risks of major adverse cardiovascular and cerebrovascular events by adjusting antiplatelet regimens [8]. Moreover, catechol-o-methyltransferase (COMT) Val158Met polymorphism (rs4680) is involved in pain modulation, which has been evidenced to influence opioid use in pediatric patients with cancer and adult patients with chronic noncancer pain [9,10]. It seems PGx testing of COMT polymorphism can provide a reference for adjusting the dosage of opioids to achieve satisfactory pain relief while reducing adverse drug reactions.

There is great diversity in how PGx testing is applied across countries. Since the United States and the European countries were among the first to apply PGx testing in clinical practices, they have accumulated a wealth of knowledge about and experience with PGx testing. Previous research revealed that physicians in these countries generally held a positive attitude towards making treatment decisions that were informed by PGx testing results [11,12]. As physicians are those who order PGx testing and prescribers of medications, they are key to providing personalized medical services for patients. They should have an overall understanding of PGx testing and the capacity to order appropriate PGx testing for patients as well as optimize treatment regimens according to PGx testing results. In China, the National Health and Family Planning Commission of China (NHFPC) published two guidelines on PGx testing and its application in clinical practices early in 2015. However, the uptake of PGx testing in China remains slow, and a significant number of PGx tests recommended by the guidelines are still largely restricted to laboratory research. So far, how Chinese physicians perceive PGx testing and their capacity to use such tests in practice remain poorly understood. Therefore, the aim of this survey was to explore physicians’ knowledge of, attitudes towards, and perceived capacity as well as obstacles regarding PGx testing in China.

## 2. Materials and Methods

### 2.1. Survey Development

This research is a cross-sectional investigation. An online survey was developed based on previous literature [13,14,15,16,17,18,19,20] and knowledge of PGx testing in China. The initial survey was reviewed by four experts in the field of PGx before formal dissemination. The final survey contained 39 questions with an estimated 20 min to complete (Appendix A). Questions were divided into five sections as follows: (1) participant characteristics; (2) knowledge about PGx testing and confidence in personal capacity to order PGx testing; (3) attitudes toward the use of PGx testing in routine clinical practices, (4) practical experience of ordering PGx testing; and (5) perceived obstacles to increasing the uptake of PGx testing and preferred sources to learn about PGx testing. The perceived knowledge, self-assessed competencies, general attitude, and contributing factors of practice were measured on an integer scale of five, and the perceived value of PGx testing application in different treatment areas among physicians was measured on a percentile scale. The primary outcomes included the score of physicians’ perceived knowledge, general attitude, and confidence in personal capacity regarding PGx testing. At the beginning of this questionnaire, the respondent was asked to sign an informed consent. If the respondent selected “I volunteer to participate in the study”, all questions should be answered.

### 2.2. Survey Population

We recruited the targeted participants with the online medical platforms for chronic disease management and health service, MEDLINKER (https://www.medlinker.com/) (accessed on 28 August 2022) and “Dazhuanjia” (http://www.dazhuanjia.com/) (accessed on 28 August 2022). The two platforms had more than 80 million real-name registered physicians covering cardiovascular and cerebrovascular, oncology, psychiatry, respiratory, gastroenterology, endocrinology, nephrology, pediatrics, and so forth. Moreover, their working seniority, departments, and medical institutions were comprehensively distributed, which provided a suitable group of physicians for survey in this study. Physicians who were qualified based on the following recruitment criteria were invited to participate in the survey: (1) worked as a doctor in any department with a practicing medical license; (2) practiced in formal medical institutions, which included public hospitals at all levels and community medical institutions; and (3) located in the capital cities and sub-provincial cities of 23 provinces, the capital cities of 5 autonomous regions, 4 municipalities directly under the Central Government, and 2 special administrative regions.

Qualified physicians were sent an invitation with a brief introduction of our survey and those who expressed an interest to participate in the survey would receive the complete electronic questionnaire. The questionnaire was set with “prohibiting duplicate answers” and there was only one chance for any IP address to fill out the questionnaire. The first invitation was released on 10 October 2021 and the questionnaire was open for 95 days until 12 January 2022. Questionnaire distribution and information collection were completed through the professional survey platform “Wenjuanxing” (https://www.wjx.cn, accessed on 28 August 2022). All data are anonymous and aggregated, meaning individual respondents cannot be identified. The study was approved by the Institutional Review Boards at Peking University, Beijing, China (IRB 2021100). Respondents who finished the questionnaire completely would receive 10 yuan (~US$1.48) as a reward.

### 2.3. Data Analysis

We used STATA (version 16.0) to clean, transfer, integrate, and analyze all data obtained from collected surveys. Data were summarized using descriptive statistics. Frequencies and proportions were used to describe categorical outcomes. The measurements of central tendency and dispersion were expressed for continuous outcomes. After testing the applied prerequisites and conditions of statistical methods, *t*-test, one-way ANOVA, chi-square, and Kruskal–Wallis H test were selected to compare differences in items related to PGx testing by participant characteristics. All hypothesis tests were two-tailed. A p-value of less than 0.05 was considered statistically significant.

## 3. Results

### 3.1. Respondent Characteristics

A total of 450 physicians completed the questionnaire, and 366 of them were deemed eligible for study inclusion based on the first criterion but those who did not work as a doctor anymore were excluded, leaving a survey completion rate of 81.3%. Among the 366 physicians included in the study, 275 (75.1%) had heard of PGx testing before participation (who will be referred to as the pre-knowns hereafter) while 91 (24.9%) had not. Table 1 provides participant characteristics. No significant differences were detected in whether the physicians have heard of PGx testing by gender (*p* = 0.277), the experience of studying abroad (*p* = 0.083), and the GDP level of the province whether the participant practiced (*p* = 0.594). Age group (*p* = 0.000), highest educational attainment (*p* = 0.000), work seniority (*p* = 0.000), professional title (*p* = 0.000), hospital level (*p* = 0.000), the relationship between the hospital and a medical college (*p* = 0.001), and their attitude to new technology (*p* = 0.000) were significantly associated with whether they had heard PGx testing. The following results are based on surveys collected from the pre-knowns.

Of the 275 pre-knowns, more than half were men (56.0%) and most fell into the age groups of 26–35 years old (31.6%) or 36–45 years old (44.0%). Many had the highest educational attainment of a bachelor’s degree or below (57.5%), followed by a master’s degree (32.4%) and a doctoral degree (10.2%). The majority of them had never studied abroad (92.4%) and only a few physicians had exchanged (6.2%) or pursued a degree program (1.5%) abroad. More than half of the pre-knowns had worked as a physician for more than 10 years (53.5%), followed by less than 5 years (27.3%), and 6–10 years (19.3%). When categorized by professional titles (from high to low), 13.5% were chief physicians, 29.8% were associate chief physicians, 37.8% were attending doctors, and 18.9% were resident physicians. More than half (63.3%) worked in tertiary hospitals, followed by secondary hospitals (25.8%), other types of formal medical institutions (6.9%), and primary hospitals (4.0%). The pre-knowns primarily worked in 27 provinces in mainland China, which could be further divided into high-GDP provinces (82.2%) and middle-low-GDP provinces (17.8%).

Considering that respondents’ willingness to adopt new technologies might influence their attitude towards PGx testing, a relevant question was included in the questionnaire. Most pre-knowns (74.6%, n = 205) were neutral about adopting new technologies and selected “aware of the need to change and very comfortable adopting new technologies and would adopt new technologies before the average person but need to see evidence of success before adopting”. Meanwhile, 60 (21.8%) and 10 (3.6%) respondents chose “want to be the first person to try an innovation” and “skeptical of change, only adopt an innovation after it has been tried by the majority”, respectively.

### 3.2. Physicians’ Knowledge and Confidence of PGx Testing

When physicians were asked about their perceived knowledge about PGx testing, most (61.5%) rated it as “Fair”. Only 20.0% chose “Excellent” or “Good”, and 18.6% chose “Poor” or “Terrible”. Significant differences were observed in physicians’ perceived knowledge across different professional titles (associate chief vs. resident: *p* = 0.04, OR = 0.37), highest education attainment (doctoral degree vs bachelor’s degree or below: *p* = 0.005, OR = 0.24), attitudes to new technology (neutral attitude vs. positive attitude: *p* = 0.005, OR = 0.24), and whether the hospital had a dedicated person to provide personalized pharmaceutical care based on PGx testing results (not provided vs. provided: *p* = 0.035, OR = 1.95).

**Figure 1:**
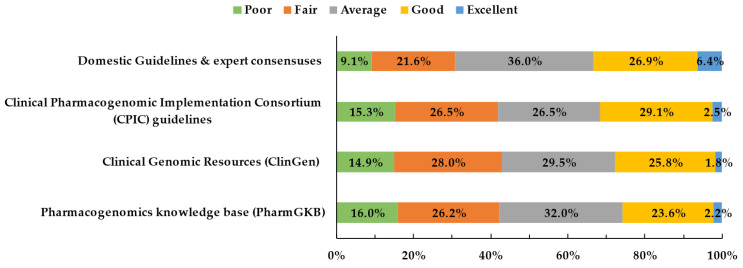
Physicians’ knowledge of resources related to PGx testing.

Physicians’ knowledge of resources related to PGx testing is illustrated in Figure 1. Participants generally had poor knowledge about resources related to PGx testing and less than 10% were knowledgeable about relevant guidelines or databases. More than 40% of the physicians thought their knowledge about CPIC guidelines, Clinical Genomic Resources (ClinGen), and Pharmacogenomics knowledge base (PharmGKB) were below average. More physicians believed that they had comprehended domestic guidelines and expert consensuses well (6.4%) than foreign ones.

**Figure 2:**
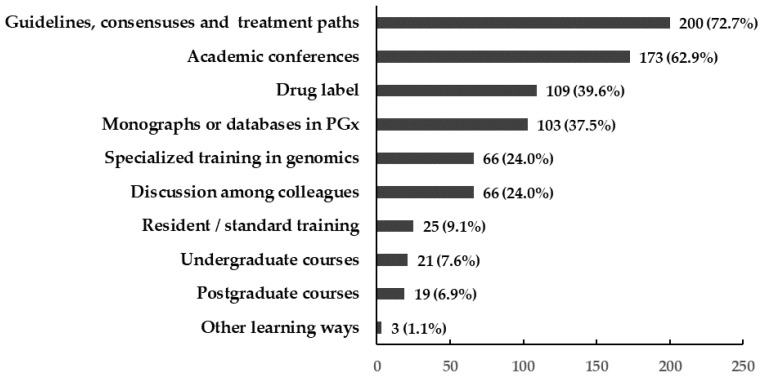
Physicians’ ways of learning information about pharmacogenomic testing.

The most common sources for physicians to learn about PGx testing were guidelines, consensuses, and treatment paths for diseases (72.7%); academic conferences (62.9%); drug labels (39.6%); monographs and databases in pharmacogenomics (37.5%); and so forth (Figure 2).

The average for physicians’ confidence in their personal capacity regarding PGx testing was 3.30 out of 5.00, with a standard deviation of 0.09 (Table 2). Respondents were most confident in deciding which drugs needed PGx (3.42 ± 0.08) and tailoring treatment and monitoring regimens based on the results of PGx testing (3.40 ± 0.09).

### 3.3. Attitudes towards PGx Testing among Physicians

**Figure 3:**
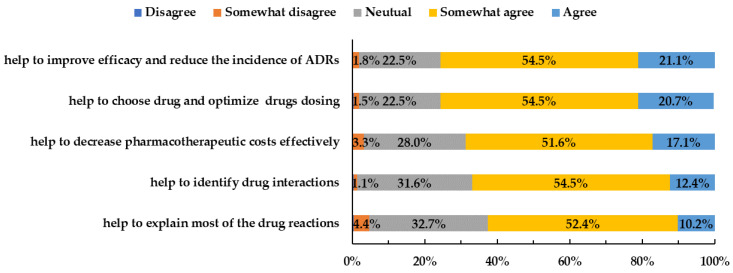
Physicians’ attitudes toward PGx testing.

Physicians generally acknowledged the significance of PGx testing. Most respondents agreed that PGx testing could “help to improve efficacy and reduce the incidence of adverse reactions from drug therapy” (75.6%) and “help to choose drugs and optimize drug dosing” (75.2%). For all questions, more than half agreed with the description presented (Figure 3).

Attitudes towards PGx achieved an average total score of 72.87 out of 100.00 with a standard deviation of 1.00 (Table 3). Among the six specific treatment areas outlined, targeted cancer therapy (78.95 ± 1.26) gained the highest score, with more than half of respondents rated the value of PGx as 80–100 in this therapeutic area.

### 3.4. Physicians’ Experience with PGx Testing

Only 124 physicians (45.1%) were confident about the availability of PGx testing at their practicing institution. Hospitals most commonly provided PGx testing for the purposes of clinical diagnosis and treatment as well as scientific research (n = 85, 68.5%). Some physicians (n = 35, 28.2%) stated that their hospital provided PGx testing only for diagnosis and treatment purposes.

More than half (n = 154, 56.0%) had ordered PGx testing before, and they most frequently ordered tests for patients (91.6%), followed by colleagues (47.4%), patients’ family (37.7%), their family members (3.9%), and themselves (3.9%). Among participants who had ordered PGx testing, more than half had ordered PGx tests for 1 to 10 cases a year (n = 90, 58.4%). Others had ordered for 11 to 20 cases a year (13.0%), for 21 to 30 cases a year (10.4%), or for over 30 cases a year (10.4%).

**Figure 4:**
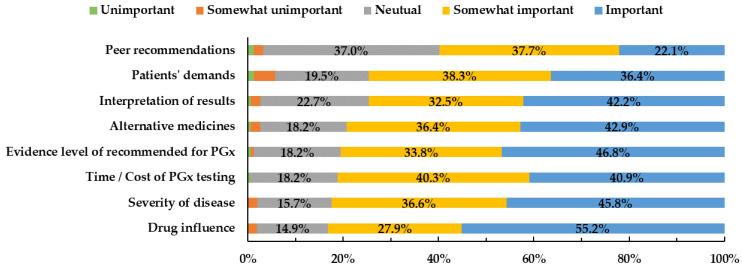
Factors contributing to physicians’ PGx prescription.

Several factors affected if a physician ordered PGx tests (Figure 4). “The extent to which PGx testing results affect drug efficacy or adverse effects” (83.1%), “severity of disease” (82.4%), “time/cost of PGx testing” (81.2%), and “evidence level of recommended for PGx testing” (80.6%) were the four most important factors for doctors to consider when deciding whether to order or recommend PGx testing.

A total of 162 physicians (58.9%) had the experience of interpreting PGx results to their patients. PGx testing results impacted physician behavior in a number of ways (see Figure S1). The majority (84.6%) pointed out that “If the result of pharmacogenomic testing is relevant to drug efficacy, I will adjust the drug option” and another three solutions for drug adjustment were also chosen by about half of the physicians. Only one respondent refused to adjust the drug regimen based on the results of PGx testing. References that physicians used to interpret the results of PGx testing are shown in Figure S2. Relevant guidelines or documents (77.8%) and drug labels (74.7%) were the primary references to consult for physicians.

### 3.5. Perceived Obstacles and Educational Preferences

Perceived obstacles to increasing the uptake of PGx testing included lack of PGx professionals (67.6%), high costs of PGx testing (61.8%), lack of PGx knowledge (60.4%), and lack of hospitals or institutions to provide PGx service (58.2%) (see Figure S3). Among all respondents, 294 (80.3%) showed a willingness to participate in training related to PGx if provided, while 33 (9.0%) were not willing. For the 294 respondents who were willing to participate in such training, the most popular training modes were academic conferences (72.4%), expert lectures (69.0%), and online video courses (68.0%) (see Figure S4).

## 4. Discussion

Our survey is the first one to evaluate physicians’ perspectives on PGx and its clinical implementation in China. Our findings highlight important concerns about the discrepancies between physicians’ perceived values of PGx testing and their knowledge about and capacity to order such tests in China.

The survey indicated that nearly three quarters of the respondents had heard of PGx testing before being involved in this survey, which is similar to the findings of a survey of American primary care physicians by Susanne et al. [14]. Higher percentages of physicians were exposed to PGx testing than in the research of Kuwait [20], but lower percentages of physicians were personally involved in PGx practices than that reported in other research in the USA [11,12]. Moreover, less than half of physicians pointed out that the hospitals where they worked provided PGx testing. A total of 75.1% of the physician respondents had heard of PGx testing, a lower percentage than that for clinical pharmacists (99.1%) in China [21]. This may indicate that clinical pharmacists are willing to put more effort and energy into integrating PGx testing into clinical practice than physicians, which is consistent with the findings in Qatar, Kuwait, and Malaysia [20,22,23]. Moreover, previous studies suggested that physicians, as well as other healthcare professionals, also have supported pharmacists to provide PGx services rather than physicians [24,25,26]. This may be due to the fact that by having knowledge of drug metabolism pathways and drug–gene interactions, pharmacists can play a significant role in PGx education, implementation, and provision [27], which is also supported by the American Pharmacists Association (APhA) [28].

Physicians who participated in our survey generally had poor knowledge about resources relevant to PGx testing. Even if they had experience with PGx testing before, not all of them were ready to make clinical decisions based on PGx results. Physicians also considered patient needs and the quality of evidence related to PGx testing when deciding whether to order or recommend PGx tests. Guidelines, pertinent literature, and drug instructions were common sources for physicians to learn about PGx and interpret results. This suggests that Chinese physicians pay little attention to relevant guidelines or databases, which may also be among reasons why PGx testing results has hardly been used to guide clinical decision-making. Surveys in the U.S. have reported similar results that physicians had an inadequate understanding of available resources to consult about PGx testing [17,29,30]. Chinese physicians in our survey were more familiar with domestic guidelines and monographs than foreign ones, which is consistent with what we have found among clinical pharmacists [31]. Moreover, clinical pharmacists in our previous survey knew more about domestic guidelines (50.9% chose good or excellent) than physicians. Given that there are only six domestic consensuses or guidelines in China relevant to PGx testing, inviting experts in PGx to develop guidelines and databases seems to be imminent and would help medical professionals to adopt PGx testing.

Physicians scored themselves higher than clinical pharmacists (mean 5.12 ± 3.09, scale 0–10) on the competencies related to PGx testing, which reflects that physicians were generally more confident in their capacity to provide PGx services than pharmacists, although they have a similar grasp of PGx testing. Physicians in our study were more confident in identifying drugs that require PGx testing and applying the results of PGx testing to deciding drugs, drug dosing, or monitoring, which is in concordance with previous research [20,22,23,32]. Physicians can modify drug regimens for patients based on the results of PGx testing more directly than pharmacists, who are better positioned to interpret PGx results and provide relevant information. Our survey indicated that a positive attitude towards new technologies in general and towards the use of PGx testing can prompt physicians to participate in relevant training, but their perceived knowledge and confidence in PGx testing did not influence their willingness to receive training, which echoes the study by Teresa et al. [33]. Physicians’ positive attitudes toward PGx testing contrasted with their lack of confidence to provide PGx information. Therefore, it is important for medical professionals to provide patients with efficient PGx testing services collaboratively.

Physicians valued PGx testing the most in targeted cancer therapy, which is related to the current trend of applying PGx testing in molecularly targeted agents [34]. However, it is worth noting that physicians’ expectations for PGx testing in neurological and psychiatric diseases were far lower than in other clinical fields. This might also be related to the fact that few psychiatrists were included in our survey and that the application of PGx testing in psychiatry and neurology remains limited, with some PGx clinical trials for neurological and psychiatric diseases underway [35].

A higher proportion of physicians (56.0%) had prescribed PGx testing in our study. In addition, 58.9% of physicians had interpreted PGx results for their patients, which was similar to the involvement of clinical pharmacists (59.0%). However, physicians and clinical pharmacists differed in how often they were involved in PGx testing. More than half of physicians (58.4%) ordered PGx testing for 1 to 10 cases per year, while only 10.4% ordered PGx testing for more than 30 cases per year. By contrast, fewer clinical pharmacists (41.0%) provided PGx-relevant services at a rate of 1 to 10 cases per year, and more clinical pharmacists (21.9%) provided services for more than 30 cases per year, which revealed that clinical pharmacists participated in PGx services more frequently and might have accumulated richer experience [31]. This may be due to the differences in division of PGx testing between physicians and clinical pharmacists. In China, clinical pharmacists can serve as subject matter experts in clinical PGx and assume three different roles in PGx practice: researcher, educator, and clinical practitioner [28]. Clinical pharmacists are more interested in participating in PGx testing and related services than physicians, and they believe that clinical pharmacists should take on the role of “making recommendations to physicians on drug selection, dosage, and monitoring based on the results of PGx testing” and “Interpret PGx testing results to physicians and patients”. Moreover, the physicians in this study also pointed out that the lack of professional interpretation of the results of PGx limits the widespread use of PGx in clinical practice, and pharmacists’ suggestions were one of the common PGx information sources for them. This highlights the need for clinical pharmacists to assume responsibility for PGx, including encouraging the optimal use of PGx tests, interpreting the findings of PGx tests, and educating healthcare professionals and patients about the information of PGx [28]. Therefore, in the face of the current situation that the role of clinical pharmacists in PGx-related work is not fully exploited due to the lack of confidence in their competence to deliver services, there is an urgent need to strengthen the training for different types of healthcare professionals.

It is interesting to note that the perceived obstacles to increasing the uptake of PGx testing in our survey are different from the results reported in a previous Chinese study, which found that the lack of standards for clinical application, large-scale clinical trials, and a Chinese guideline on the application of PGx testing were major factors hindering the uptake of PGx testing [36]. Instead, physicians included in our survey were more worried about the lack of professionals and knowledge, high costs of testing, and lack of hospitals to offer PGx testing, similar to the findings in other regions [22,30,37]. Physicians’ concerns about the lack of professionals and knowledge reinforce our view that Chinese physicians currently have difficulty meeting the demand to provide comprehensive PGx services to patients due to lack of relevant competencies and experience. Currently in China, only PGx testing for targeted cancer therapy performed in hospitals is covered by health insurance in some cities. Physicians may thus consider the testing cost as a vital factor in deciding whether to order PGx testing. In addition, our results revealed that only those hospitals with a high capacity for disease diagnosis and treatment in China provided PGx testing. As the uptake of PGx testing increases in China, the challenges to overcome include clinical trial evidence, Chinese guidelines and standards on the application of PGx testing, and training and educating medical personnel so they are better equipped with knowledge and competencies relevant to PGx testing.

Academic conferences and online video courses were the most popular modes of training among physicians. However, among clinical pharmacists, short-term intensive training (57.3%) and online video courses (56.2%) were preferred. These findings indicate that relevant academic conferences are just one feasible training method for physicians to obtain information about PGx. Online video courses may be preferred for their convenience and spread, which can be developed to provide physicians with up-to-date learning about PGx. Moreover, this also validates our previous recommendation that different training modes and content should be designed for clinical pharmacists and physicians.

In the future, while continuing to promote the uptake of PGx testing in clinical practices and improve the evidence related to PGx testing, the differences between physicians and clinical pharmacists should be considered. Physicians generally express a positive attitude towards and full confidence in PGx, but their knowledge and practice experience of PGx are not as rich as those of clinical pharmacists. Only when physicians have a better understanding of PGx and its related resources, and with the assistance of clinical pharmacists, they will have greater interest and confidence in clinical decision-making based on PGx testing.

## 5. Limitation

Our survey has some limitations. The participants in this survey were recruited from the online medical platform and all the physicians worked in different hospitals from most provinces around the country, which meant that the results of our survey were of certain generalizability in China. On the other hand, it also brought a limitation that our physicians who were willing to use online medical platforms might be likely to adopt new technology more actively and participate in PGx testing and related services. Another limitation of this survey is that the number of physicians included in our sample was relatively small, and those physicians who had heard of or been involved in PGx testing and related services before the survey were more likely to participate in our study. Thus, this would overstate physicians’ knowledge of PGx in China. We will refine this survey and conduct more in-depth studies with larger sample volumes in the future, which can reflect Chinese physicians’ involvement in PGx testing more representatively.

## 6. Conclusions

In conclusion, physicians in China perceived themselves to have poor knowledge about but strong confidence in and positive attitudes toward PGx testing. Lack of professionals and knowledge, high costs of testing, and lack of hospitals to offer PGx testing were identified as the primary obstacles to increasing the uptake of PGx testing in China. Academic conferences were regarded the most efficient way for physicians to obtain information about PGx.

## Figures and Tables

**Table 1:** Characteristics of respondents in our survey.

	All Respondents ^1^	Respondents Who Have Heard of PGx Before
	366	275
Gender		
Male	199 (54.4%)	154(56.0%)
Female	167 (45.6%)	121 (44.0%)
Age		
≤25	29 (7.9%)	12 (4.4%)
26-35	123 (33.6%)	87 (31.6%)
36-45	142 (38.8%)	121 (44.0%)
46-50	35 (9.6%)	25 (9.1%)
>50	37 (10.1%)	30 (10.9%)
Highest Educational Qualification		
Bachelor’s degree or below	235 (64.2%)	158 (57.5%)
Master’s degree	102 (27.9%)	89 (32.4%)
Doctoral degree	29 (7.9%)	28 (10.2%)
Experience of Study Abroad		
Never studied abroad	344 (94.0%)	254 (92.4%)
Studied for a short-term program	18 (4.9%)	17 (6.2%)
Studied for a degree	4 (1.1%)	4 (1.5%)
Work Seniority		
<5 year	126 (34.4%)	75 (27.3%)
6–10 years	70 (19.1%)	53 (19.3%)
10–30 years	148 (40.4%)	128 (46.6%)
>30 years	22 (6.0%)	19 (6.9%)
Professional Title		
Chief physician	40 (10.9%)	37 (13.5%)
Associate chief physician	90 (25.1%)	82 (29.8%)
Physician-in-charge	126 (34.4%)	104 (37.8%)
Resident physician	108 (29.5%)	52 (18.9%)
Level of Hospital		
Tertiary hospital	197 (53.8%)	174 (63.3%)
Secondary hospital	97 (26.5%)	71 (25.8%)
Primary hospital	23 (6.3%)	11 (4.0%)
Other types of medical institutions	49 (13.4%)	19 (6.9%)
Relationship between Hospital and a Medical College		
Affiliated hospital	149 (40.7%)	122 (44.4%)
Teaching hospital	67 (18.3%)	56 (20.4%)
Neither affiliated nor teaching hospital	150 (41.0%)	197 (35.3%)
GDP Level of the Province of Practice Location ^3^		
Middle-low GDP	63 (17.2%)	49 (17.8%)
High GDP	303 (82.8%)	226 (82.2%)
Physicians’ Willingness to Adopt New Technology ^2^		
Positive	72 (19.7%)	60 (21.8%)
Neutral	270 (73.8%)	205 (74.6%)
Negative	24 (5.6%)	10 (3.6%)

^1^ All respondents refer to those who agreed to participate in our survey and accomplished the whole questionnaire. ^2^ Positive willingness refers to the option “Want to be the first person to try an innovation”; neutral willingness refers to the option “Aware of the need to change and very comfortable adopting new technologies and adopt new technologies before the average person, but need to see evidence of success before adopting”; negative willingness refers to the option “Skeptical of change, only adopt an innovation after it has been tried by the majority”. ^3^ Provinces where the physicians practiced were sectioned into two groups according to Gross Domestic Product (GDP) rank of the provinces within the country: (1) provinces with middle-low GDP, which included Anhui (AH), Beijing (BJ), Chongqing (CQ), Guangxi (GX), Guizhou (GZ), Hebei (HeB), Jiangxi (JX), Liaoning (LN), Shaanxi (S3X), Yunan (YN), Gansu (GS), Hainan (HaiN), Heilongjiang (HLJ), Jilin (JL), Neimenggu (NMG), Ningxia (NX), Qinghai (QH), Shanxi (S1X), Tianjin (TJ), Xinjiang (XJ), and Xizang (XZ); and (2) provinces with high GDP, spanning Fujian (FJ), Guangdong (GD), Henan (HeN), Hubei (HuB), Hunan (HuN), Jiangsu (JS), Shandong (SD), Shanghai (SH), Sichuan (SC), and Zhejiang (ZJ). The data of GDP of each province were from the report published in 2020 by the Office for National Statistics (https://data.stats.gov.cn/easyquery.htm?cn=E0103, accessed on 28 August 2022).

**Table 2:** Physicians’ confidence in personal capacity regarding PGx testing.

**Items of Capability Self-Assessment**	**Mean Score** ** ± SD ** **^1^**
Evaluate which drugs need pharmacogenomic testing	3.42 ± 0.08
Tailor treatment and monitor regimes based on pharmacogenomic results	3.40 ± 0.09
Recommend pharmacogenomic testing	3.30 ± 0.09
Determine which PGx testing are available at your health care facility	3.23 ± 0.09
Interpret the results of pharmacogenomic testing	3.20 ± 0.09

^1^ SD is the abbreviation of standard deviation.

**Table 3:** Value of PGx testing in different treatment areas (scale 0–100).

Areas	Medium	0–20	20–40	40–60	60–80	80–100
Targeted cancer therapy	78.95 ± 1.26	7 (2.6%)	9 (3.3%)	39 (14.2%)	74 (26.9%)	146 (53.1%)
Cardiovascular diseases	73.41 ± 1.12	3 (1.1%)	11 (4.0%)	63 (22.9%)	103 (37.5%)	95 (34.6%)
Rheumatic diseases	73.30 ± 1.22	5 (1.8%)	15 (5.5%)	61 (22.2%)	89 (32.4%)	105 (38.2%)
Infectious diseases	71.02 ± 1.33	10 (3.6%)	15 (5.5%)	70 (25.5%)	88 (32.0%)	92 (33.5%)
Pain management	70.64 ± 1.21	4 (1.5%)	17 (6.2%)	70 (25.5%)	106 (38.6%)	78 (28.4%)
Neurological and psychiatric diseases	69.89 ± 1.23	6 (2.2%)	18 (6.6%)	71 (25.8%)	106 (38.6%)	74 (26.9%)

## Data Availability

Not applicable. Data cannot be shared publicly because of signed Sharing Data Confidential Agreements.

## Supplementary Materials

The following supporting information can be downloaded at: https://www.mdpi.com/article/10.3390/jpm12122021/s1, Figure S1: The Impact of PGx Results on Physicians Behavior; Figure S2: References to Consults When Interpreting PGx Results; Figure S3: Perceived Obstacles to Increasing Uptake of PGx Testing; Figure S4: Preferred Training Modes Related to PGx Testing.

## Appendix A

Questionnaire for Physicians’ Knowledge, Attitude, and Experience of Pharmacogenomic Testing.

Dear physician:

We are the Pharmacogenomics Research Team of Peking University. We sincerely hope to understand your views on the clinical application of pharmacogenomic testing and would like to invite you to participate in this questionnaire survey. The survey results do not contain any of your identity information, and the survey results will be kept strictly confidential and used only for academic research and not for any commercial purpose.

Please answer according to your actual situation and attitude. If you have any questions about the requirements of the questionnaire, please feel free to send them to PKUPharmageno@163.com. Thank you for your support and cooperation.

(It will take you about 10 min to complete this questionnaire.)

0. Do you agree to participate in this survey?

○YES

○NO (end)

**Part 1: Basic information**

1. What is your gender? ○Male ○Female

2. What is your age? ○≤25 ○26-30 ○31-35 ○36-40 ○41-45 ○46-50 ○51-55 ○56-60 ○61-65 ○>65

3. What is your degree and major at each level? ○Lower than Bachelor’s degree ○Bachelor’s degree ○Master’s degree ○Doctoral degree

4. Have you ever studied abroad? ○No (please skip to question 6) ○Yes (for a degree) ○Yes (for an exchange study)

5. Where did you study abroad? ○UK ○UA ○Japan ○Australia ○Canada ○Others: _________________ *

6. Which province/autonomous region/municipality do you practice in?

○Gansu (GS) ○Hainan (HaiN) ○Heilongjiang (HLJ) ○Jilin (JL)

○Neimenggu (NMG) ○Ningxia (NX) ○Qinghai (QH) ○Shanxi (S1X)

○Tianjin (TJ) ○Xinjiang (XJ) ○Xizang (XZ) ○Anhui (AH) ○Beijing (BJ)

○Chongqing (CQ) ○Guangxi (GX) ○Guizhou (GZ) ○Hebei (HeB)○Jiangxi (JX)

○Liaoning (LN) ○Shaanxi (S3X) ○Yunan (YN) ○Fujian (FJ)

○Guangdong (GD) ○Henan (HeN) ○Hubei (HuB) ○Hunan (HuN)

○Jiangsu (JS) ○Shandong (SD) ○Shanghai (SH) ○Sichuan (SC) ○Zhejiang (ZJ)

7. The name of your medical institution is: _______________ (fill in the blank)

8. How many years have you worked? ○Still in training ○<1 year ○1 year–5 years ○5 year–10 years ○10 year–30 years ○>30 year

9. The level of the hospital you work for: ○Tertiary hospital ○Secondary hospital ○First hospital ○Other: _________________

10. The relationship between your hospital and medical college is: ○Affiliated Hospital ○Teaching hospital ○Neither affiliated nor teaching hospital ○Other: _________________

11. What is your technical title? ○Resident physician ○Physician-in-charge ○Deputy chief physician ○Chief physician ○Others: _________________

12. Your department is:

○Cardiovascular Dep ○Cerebrovascular and Neurology Dep ○Oncology Dep

○Psychiatry Dep ○Digestion Dept ○Respiratory Dept ○Hematology Dept

○Nephrology Dept ○Endocrine Dept ○Rheumatology Dept ○Dermatology Dept

○ICU ○Emergency ○Obstetrics and Gynecology Dept ○Pediatrics Dept

○Others: _________________

13. For new surgical approaches, new tests, and new drugs (Collectively referred to as “new technologies”) that emerge in clinical practice, which of the following description suits you best:

○Want to be the first person to try an innovation.

○Aware of the need to change and very comfortable adopting new technologies.

○Adopt new technologies before the average person, but need to see evidence of success before adopting.

○Skeptical of change, only adopt an innovation after it has been tried by the majority.

**Part two: knowledge, confidence, and attitude of drug–gene testing**

Genetic testing is a kind of medical test that can predict, diagnose a disease, or evaluate drug response. It has the following forms:

1. Diagnostic test: a test designed to diagnose or predict disease risk. For example, genetic testing for Marfan’s syndrome or Huntington’s disease.

2. Disease susceptibility test: a test with the aim of predicting the risk of future disease by detecting whether there exist genetic variations associated with the disease. For example: detection of AGT, ATR, apoE, CYP11B2, and LPL genes associated with an increased risk of coronary heart disease

3. Pharmacogenomic (PGx) testing: a test with the aim of detecting drug metabolism or target genes, so as to find out the causes of adverse drug reactions or poor drug efficacy and guide the selection of therapeutic drugs and their dosages. For example, CYP2C9 and VKORC1 genotypes can be detected to guide the application of warfarin, CYP2C19 genotypes can be detected to guide the application of clopidogrel, and CYP2D6, ADRB1, and GRK4 genotypes can be detected to guide the application of metoprolol.

14. Have you ever heard the term “pharmacogenomic testing” before you took this survey? ○YES ○NO

15. How much do you know about pharmacogenetic testing? ○Poor ○Fair ○Average ○Good ○Excellent

16. Where have you obtained/learned information about pharmacogenetic testing (multiple choice) *

○In drug labels ○In undergraduate courses ○In postgraduate courses

○In the guidelines, consensus, or standard clinical paths for disease diagnosis and treatment

○In professional guidelines, monographs, or databases ○Specialized training in genomics

○Residency training or training period ○In academic conferences

○In the discussion of colleagues ○Others: _________________

17. What extent do you know about the following pharmacogenomic-related guides, monographs, or databases?

(1) Clinical Pharmacogenomic Implementation Consortium (CPIC) guidelines

○Poor ○Fair ○Average ○Good ○Excellent

(2) Pharmacogenomics knowledge base (Pharm GKB)

○Poor ○Fair ○Average ○Good ○Excellent

(3) Clinical Genomic Resources (ClinGen)

○Poor ○Fair ○Average ○Good ○Excellent

(4) Resources such as domestic guidelines or expert consensus

○Poor ○Fair ○Average ○Good ○Excellent

18.Please self-assess your ability related to pharmacogenetic testing: (Enter the number from 0 to 10)

○I have learned which drugs need to be tested by PGx testing.

○I have the ability to tailor medicated and monitoring regimes based on PGx results.

○I have the ability to prescribe or recommend personalized PGx testing.

○I have learned which kinds of PGx testing are available in my hospital.

○I have the ability to interpret the results of PGx testing.

19. Which of the following categories of patients do you think will benefit most from pharmacogenomic testing?

○All patients ○Patients with numerous comorbidities

○Patients whose pharmacogenomic testing can guide the determination of the initial dose

○Patients who use the test result to help to predict that s/he will have a poor response to a medication

○Patients who use the test result to help to predict that s/he will experience side effects from a medication

○I do not think pharmacogenomic testing is beneficial to patients.

20. Which is true about the role of pharmacogenomic testing in your opinion?

(1) It can help to explain most of the drug reactions.

○Disagree ○Somewhat disagree ○Neutral ○Somewhat agree ○Agree

(2) It can help to identify drug interactions.

○Disagree ○Somewhat disagree ○Neutral ○Somewhat agree ○Agree

(3) It can help to choose drugs and optimize drug dosing.

○Disagree ○Somewhat disagree ○Neutral ○Somewhat agree ○Agree

(4) It can help to improve efficacy and reduce the incidence of adverse reactions.

○Disagree ○Somewhat disagree ○Neutral ○Somewhat agree ○Agree

(5) It can effectively help to decrease pharmacotherapeutic costs.

○Disagree ○Somewhat disagree ○Neutral ○Somewhat agree ○Agree

21. For patients in different areas of disease treatment, what is your opinion: (Enter the number from 0 to 100 to indicate the value of pharmacogenetic testing in assisting clinical treatment decision making)

(1) Targeted oncology therapy____________

(2) Pain treatment____________

(3) The field of psychiatry and neurology____________

(4) The field of cardiovascular disease____________

(5) The field of infectious diseases____________

(6) The field of rheumatic immune diseases____________

(7) Other fields____________

22. In your opinion, do you attach more importance to the effect of pharmacogenetic testing results on efficacy or on adverse reactions?

○Efficacy ○Adverse reactions ○Uncertain

23. What is the possibility that you will recommend/prescribe PGx testing for your patients in the future?

○Highly possible ○Generally possible ○Generally impossible ○Impossible ○Uncertain

24. Are you more likely to choose an alternative nongenetic test (e.g., a routine biochemical test) rather than a PGx testing if the nongenetic test is available to predict or assess the drug efficacy or safety?

○YES ○NO ○Uncertain

**Part three: practical experience with PGx testing**

25. Does your medical institution provide pharmacogenetic testing?

○YES ○NO ○I do not know.

26. What is the purpose of providing pharmacogenetic testing at your medical institution? (multiple choice) *

○Only for clinical diagnosis and treatment items ○Only for research items

○For both purposes ○Others ○I do not know

26. The department that offers pharmacogenetic testing in your medical institution is (multiple choice) *

○Pharmacy ○Clinical laboratory ○Others: _________________ ○I do not know.

28. Does your medical institution send patient samples to an out-of-hospital facility for pharmacogenetic testing?

○YES ○NO ○I do not know.

29. Does your medical institution have a dedicated person to provide individualized pharmaceutical care based on pharmacogenetic testing results?

○YES ○NO ○I do not know.

30. Have you ever prescribed/recommended any pharmacogenetic testing-related services? (multiple choice) *

○Yes, I have prescribed pharmacogenetic testing for myself.

○Yes, I have prescribed/recommended pharmacogenetic testing to patients.

○Yes, I have prescribed/recommended pharmacogenetic testing to colleagues or friends.

○Yes, I have prescribed/recommended pharmacogenetic testing to relatives.

○No, I have not prescribed/recommended pharmacogenetic testing to others. (Please skip to question 37)

31. On average, how often do you prescribe or recommend PGx testing per year?

○1-5 cases / year ○6-10 cases / year ○11-20 cases / year ○21-30 cases / year

○>30 cases / year ○Uncertain

32. When considering whether to prescribe/recommend PGx testing for a patient, which is true about the following factors are of importance to you?

(1) Patients’ demands ○Unimportant ○Somewhat unimportant ○Neutral ○Somewhat important ○Important

(2) Peer recommendations ○Unimportant ○Somewhat unimportant ○Neutral ○Somewhat important ○Important

(3) Severity of disease ○Unimportant ○Somewhat unimportant ○Neutral ○Somewhat important ○Important

(4) The effects of PGx testing results on efficacy or adverse reactions ○Unimportant ○Somewhat unimportant ○Neutral ○Somewhat important ○Important

(5) The evidence level of recommended for PGx testing of the drug ○Unimportant ○Somewhat unimportant ○Neutral ○Somewhat important ○Important

(6) Whether there are alternative drugs that do not need PGx testing ○Unimportant ○Somewhat unimportant ○Neutral ○Somewhat important ○Important

(7) Time and expense costs of PGx testing ○Unimportant ○Somewhat unimportant ○Neutral ○Somewhat important ○Important

(8) Whether to provide the services of result interpretation ○Unimportant ○Somewhat unimportant ○Neutral ○Somewhat important ○Important

33. What kind of PGx testing have you prescribed/recommended in your clinical practice?

○Individual PGx tests, for example: _________________ (e.g., Clopidogrel—CYP2C19)

○Multiple-gene PGx panel tests, for example: _________________ (e.g., AGENMP panel tests of antineoplastic agents, to reveal genetic differences in drug response and provide therapeutic regimens for patients and physicians.)

○I cannot remember clearly.

34. Have any patients consulted you about pharmacogenetic testing-related results performed by your hospital or other institutions?

○For both purposes ○Others ○I cannot recall it

35. Would you like to adjust the drug regimen based on the results of pharmacogenetic testing?

○If the PGx result is relevant to efficacy, I will adjust the drug option.

○If the PGx result is relevant to efficacy, I will adjust the drug dosage.

○If the PGx result is relevant to ADR, I will adjust the drug option.

○If the PGx result is relevant to ADR, I will adjust the drug dosage.

○I will not adjust the drug regimen based on the results of PGx testing.

36. When you interpret PGx test results or assist doctors to make clinical treatment decisions according to the results, what kind of resources do you usually use to obtain information about pharmacogenomics testing? (multiple choice) *

○Drug labels ○Consult relevant professional guidelines/literature reports

○Consult medical professional software or APP (medication assistant/doctor station, etc.)

○Enquire PharmGKB and other genomics knowledge base

○Promotional materials from genetic testing companies

○Popular search engines such as Baidu ○Consult your supervisor

○Consult experienced doctors ○Consult the person who provides the PGx test report

**Part four: Perceived obstacles to pharmacogenetic testing and education preference**

37. Which do you think are the barriers to the development of pharmacogenomic testing? (multiple choice) *

○Pharmacogenomic testing does not influence clinical decision making

○Lack of knowledge ○Lack of relevant professionals

○Lack of hospitals or institutions to provide testing ○No guidelines, consensus, etc.

○High cost of testing ○Insurance does not cover pharmacogenomic testing.

○Pharmacogenomic testing is too difficult for physicians to understand.

○Pharmacogenomic testing is too difficult for patients to understand.

○Other

38. If there is special training related to pharmacogenomics, are you willing to participate in it? ○Yes ○No ○Not sure

39. The type of training do you want? (multiple choice) *

○Expert lectures ○Academic conferences ○Short-term intensive training

○Spread the training over several weeks ○Online video courses ○Others
