# Supplementary material for: Physicians’ Knowledge, Attitude, and Experience of Pharmacogenomic Testing in China
Source: J Pers Med. 2022 Dec 7;12(12):2021. doi: 10.3390/jpm12122021 (PMC9783535; doi:10.3390/jpm12122021)
Supplement: Supplementary file 1 [file jpm-12-02021-s001.zip › jpm-1975867-supplementary.pdf]

Supplementary Materials

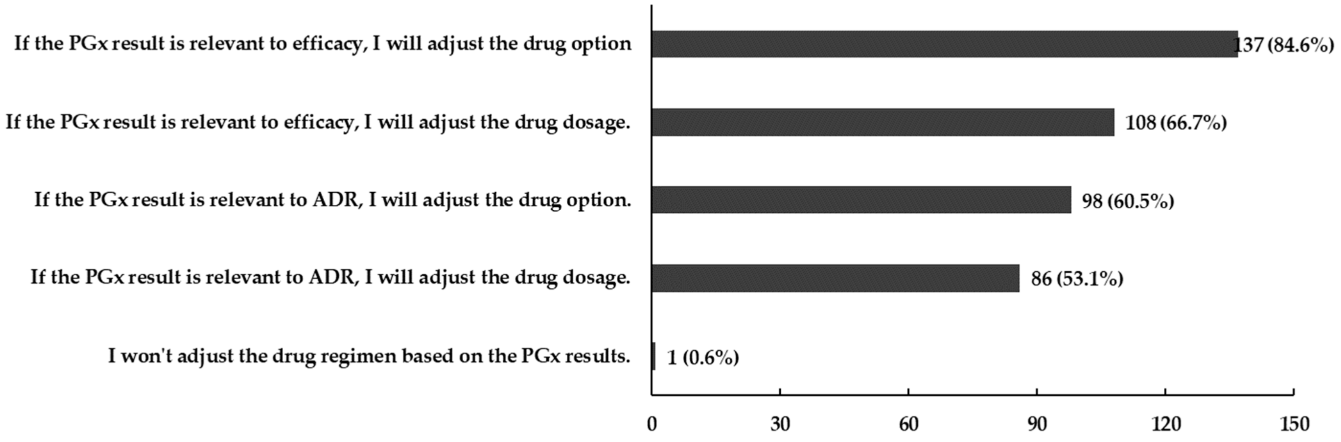

Figure S1. The Impact of PGx Results on Physicians Behavior.

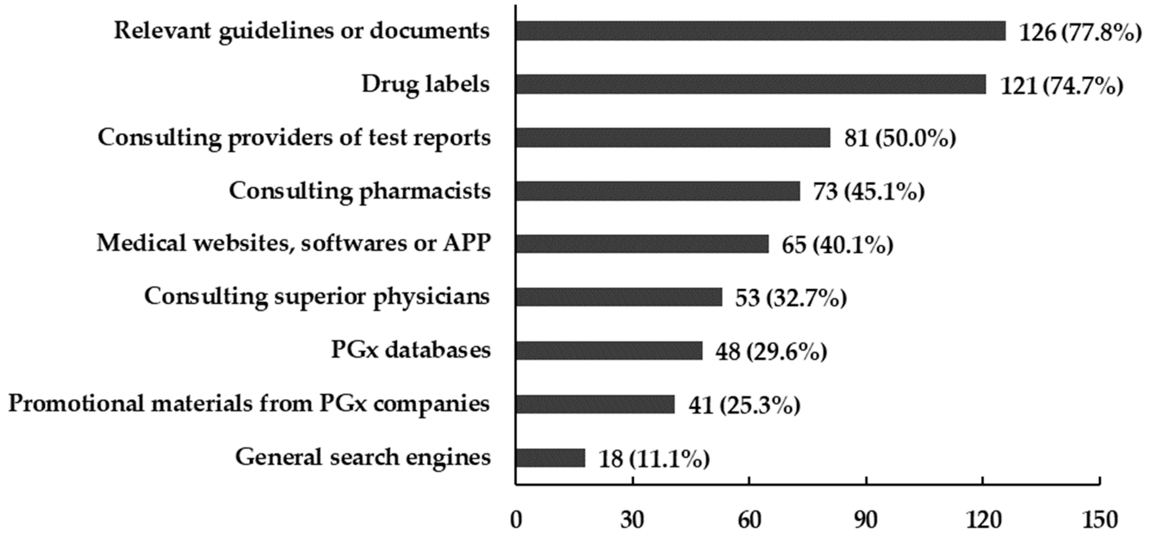

Figure S2. References to Consults When Interpreting PGx Results.

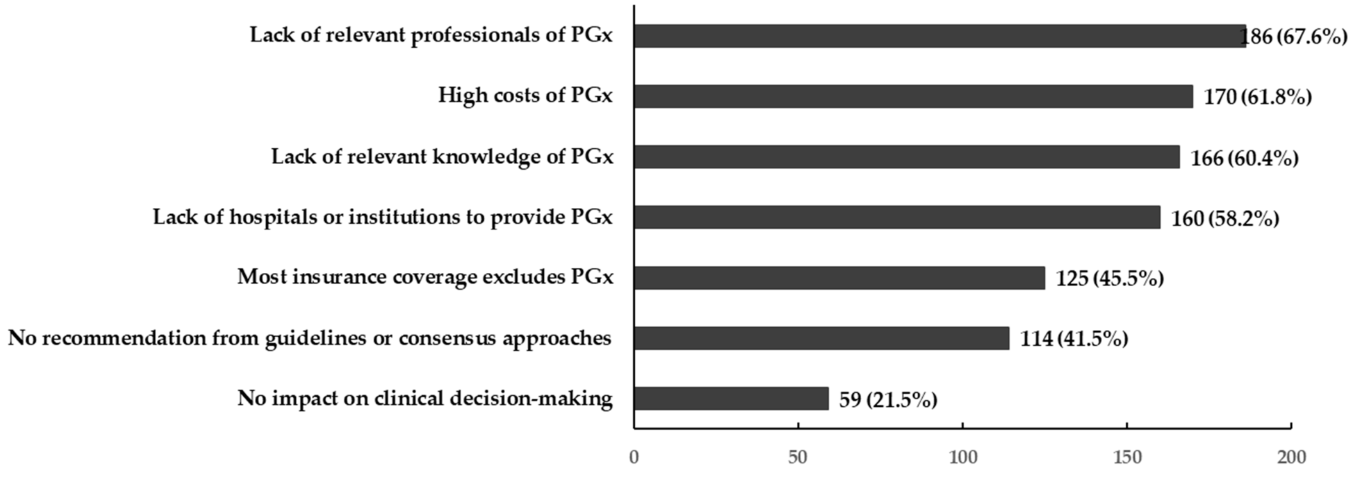

Figure S3. Perceived Obstacles to Increasing Uptake of PGx Testing.

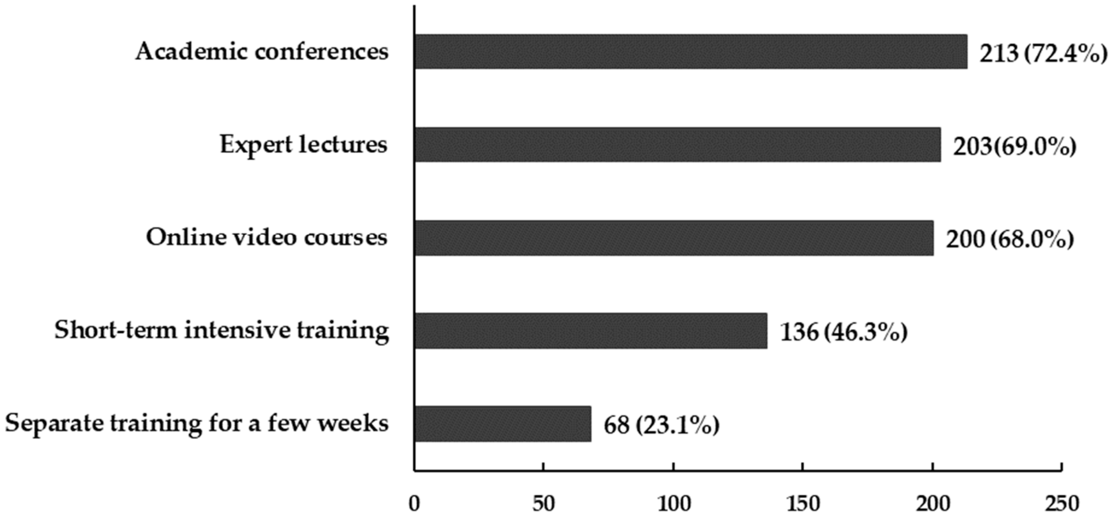

Figure S4. Preferred Training Modes Related to PGx Testing.
